# Recurrent Dislocation Following Reverse Total Shoulder Arthroplasty for Acute Proximal Humeral Fracture: Salvage With Large-Head Bipolar Hemiarthroplasty and Brachial Plexus Neuropraxia

**DOI:** 10.7759/cureus.111942

**Published:** 2026-07-02

**Authors:** Saud N Aldanyowi, Neil Ashwood, Andrew P Dekker, Paul Erdman, Amol Tambe

**Affiliations:** 1 Trauma and Orthopaedics, King Faisal University, Al Hufuf, SAU; 2 Trauma and Orthopaedics, University Hospitals of Derby and Burton National Health Service (NHS) Foundation Trust, Burton-on-Trent, GBR; 3 Trauma and Orthopaedics, Royal Derby Hospital, Derby, GBR

**Keywords:** bipolar hemiarthroplasty, brachial plexus neuropraxia, dislocation, ortho-surgery, proximal humeral fracture, reverse shoulder arthroplasty, revision arthroplasty, salvage surgery, shoulder instability, upper extremity trauma

## Abstract

Reverse shoulder arthroplasty (RSA) is commonly performed for complex proximal humeral fractures in older adults when fixation or anatomic reconstruction is unlikely to provide reliable outcomes. Recurrent instability following RSA remains a devastating complication, particularly in medically frail patients with poor bone quality and compromised soft tissue balance. Salvage strategies after repeated failed revisions are limited and often associated with poor functional outcomes. A 75-year-old man with severe chronic obstructive pulmonary disease, hypertension, hypercholesterolaemia, transitional cell carcinoma, rectal polyposis, active smoking history, and frailty sustained a comminuted, displaced left proximal humeral fracture with subglenoid humeral head dislocation following a mechanical fall. He underwent hybrid reverse-polarity total shoulder replacement. One week postoperatively, the shoulder developed anterior instability requiring closed manipulation under anaesthesia. Despite a temporary reduction, recurrent instability persisted during rehabilitation, necessitating two subsequent open revision procedures, including liner exchange with adjustment of implant inclination and later glenosphere cup modification. Persistent instability led to referral and multidisciplinary review at a tertiary shoulder centre. The failed RSA was ultimately converted to a large-head bipolar hemiarthroplasty as a salvage procedure. The hemiarthroplasty later dislocated anteriorly, with migration of the prosthetic head into the anterior deltopectoral/coracoid region and severe upper-limb dysfunction suggestive of brachial plexus neuropraxia. Given the patient’s significant comorbidities and elevated operative risk, further reconstruction was deemed unsuitable after shared decision-making, and conservative management was pursued. The patient subsequently developed septic arthritis of the contralateral shoulder, further worsening overall functional status. Serial imaging through 2025 demonstrated persistence of the dislocated bipolar implant without evidence of implant fracture or gross stem loosening. This case illustrates the catastrophic progression of recurrent instability following fracture-related RSA in a medically frail patient with compromised bone and soft tissue conditions. It underscores the importance of careful patient selection, accurate restoration of implant positioning and soft tissue tension, prompt tertiary multidisciplinary involvement after failed stabilisation, and realistic counselling regarding the limited salvage potential and persistent complications associated with hemiarthroplasty conversion after failed RSA. This case highlights that, in medically frail patients with multiple risk factors for instability, early recognition of recurrent dislocation and timely referral to specialist shoulder centres may help guide management decisions, optimise patient expectations, and avoid repeated interventions with diminishing likelihood of durable success.

## Introduction

This case is reported using a Consensus-based Clinical Case Reporting (CARE)-informed structure to improve transparency, chronological clarity, and clinical interpretability [1,2]. Reverse shoulder arthroplasty (RSA) was originally popularised through the Grammont design principles and remains a major reconstructive option when the rotator cuff, tuberosities, or proximal humeral anatomy cannot reliably support an anatomic solution [3,4].

Despite its biomechanical advantages, RSA is a demanding operation with a meaningful complication profile. Systematic reviews and revision-focused analyses consistently identify instability, infection, humeral-sided problems, glenoid failure, and neurological injury among the most clinically important adverse events [5,6]. In the acute fracture setting, RSA has become particularly attractive for elderly patients with comminuted three- or four-part proximal humeral fractures, fracture-dislocations, poor tuberosity prognosis, or pre-existing cuff insufficiency [7-10]. Instability is especially problematic because it is rarely explained by a single factor. Technical contributors include glenosphere position, baseplate inclination, humeral version, polyethylene constraint, impingement, and failure to restore deltoid tension. Patient-level contributors include frailty, previous surgery, poor bone stock, neurological disease, soft tissue incompetence, and inability to comply with protected rehabilitation [11-16].

The management of recurrent RSA dislocation is not standardised. Closed reduction may be attempted for early dislocation, but recurrent or revision-setting instability often requires open surgery, liner exchange, glenosphere upsizing or lateralisation, humeral revision, infection exclusion, or conversion to an alternative construct [12-16]. When both the glenoid and proximal humerus are severely compromised, options narrow further. Conversion to hemiarthroplasty has been described as a salvage strategy that may reduce pain and provide low-level function in selected patients, but its mechanical success depends on residual containment and soft tissue competence [17]. Neurologic complications after shoulder arthroplasty are usually uncommon but clinically consequential, and brachial plexus-level dysfunction can be devastating when the operated limb becomes globally nonfunctional [18].

This case report aims to highlight the importance of structured assessment after early RSA dislocation, including evaluation of component position, soft tissue tension, infection, neurological status, rehabilitation risk, and the limited salvage potential in frail patients with severe bone and soft tissue compromise.

## Case presentation

Patient information

A 75-year-old man presented in December 2019 after a mechanical fall at home. His relevant comorbidities included advanced chronic obstructive pulmonary disease with documented spirometric impairment, hypertension, hypercholesterolaemia, transitional cell carcinoma, rectal polyposis, active smoking history, and frailty. These comorbidities were clinically important because they increased anaesthetic risk, limited rehabilitation capacity, and narrowed subsequent salvage options. One week after surgery, during prescribed mobilisation exercises, the patient experienced a sudden audible pop associated with acute shoulder pain, raising concern for early postoperative instability.

Index Presentation and Injury (December 2019)

The patient lost his balance whilst bending to retrieve an object and fell to the floor. He denied prodromal cardiac or neurological symptoms, loss of consciousness, seizure activity, or head injury. Immediate left shoulder and chest pain were reported. On examination, he was neurologically intact with preserved motor and sensory function in all peripheral nerve distributions of the left upper limb. He was haemodynamically stable.

**Figure 1:**
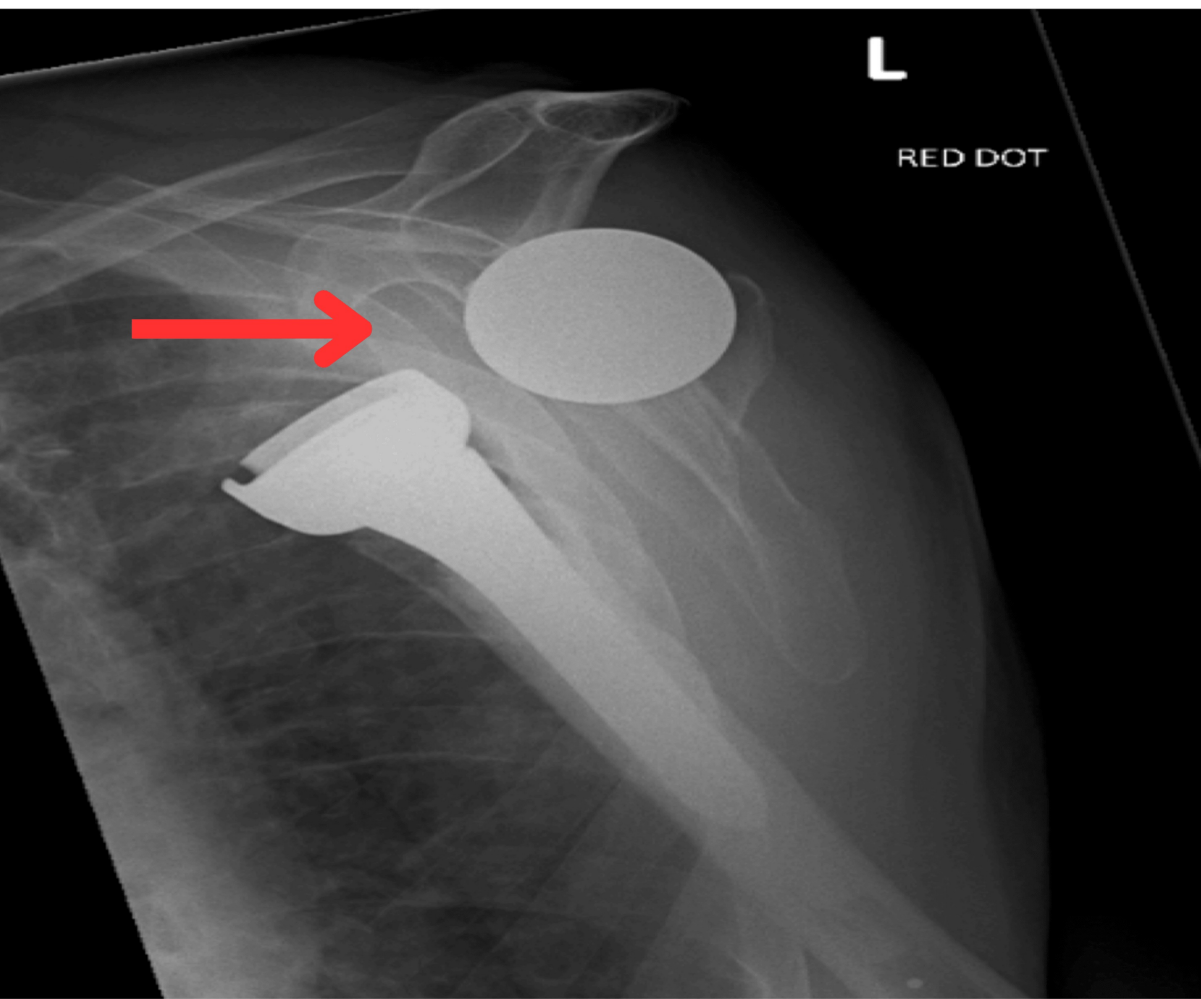
Radiograph confirming the first anterior dislocation of the reverse total shoulder replacement

Computed tomography of the thorax, abdomen, and pelvis with dedicated shoulder imaging (CT-TAP, December 2019) demonstrated a comminuted, displaced fracture of the upper end of the left humerus with dislocation of the humeral head into the subglenoid region (Figure 1). No associated visceral injury was identified. The fracture configuration was not amenable to open reduction and internal fixation, and arthroplasty was indicated.

**Figure 2:**
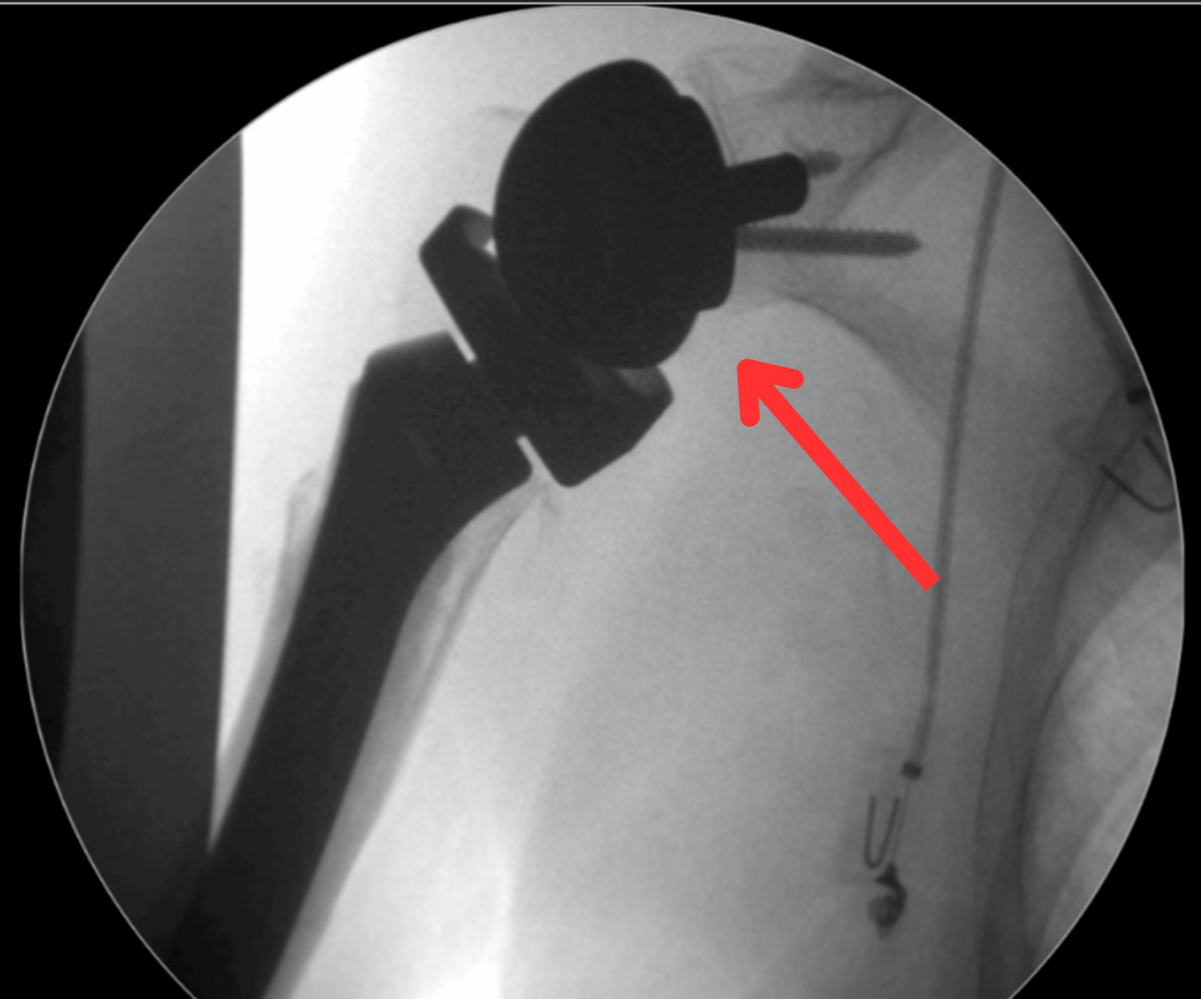
Post-MUA radiograph MUA: manipulation under anaesthesia

Following orthopaedic and anaesthetic review, the patient underwent a hybrid reverse polarity total shoulder replacement of the left shoulder in December 2019 (Figure 2), performed via the standard deltopectoral approach under general anaesthesia in the beach-chair position. The procedure was uncomplicated intraoperatively. In view of his severe chronic obstructive pulmonary disease (COPD) and difficulties maintaining oxygen saturations intra-operatively, he was transferred to the High Dependency Unit (HDU) for post-operative respiratory monitoring, stepping down to the ward after 24 hours once stable. He was commenced on intravenous piperacillin-tazobactam for hospital-acquired pneumonia. 

**Figure 3:**
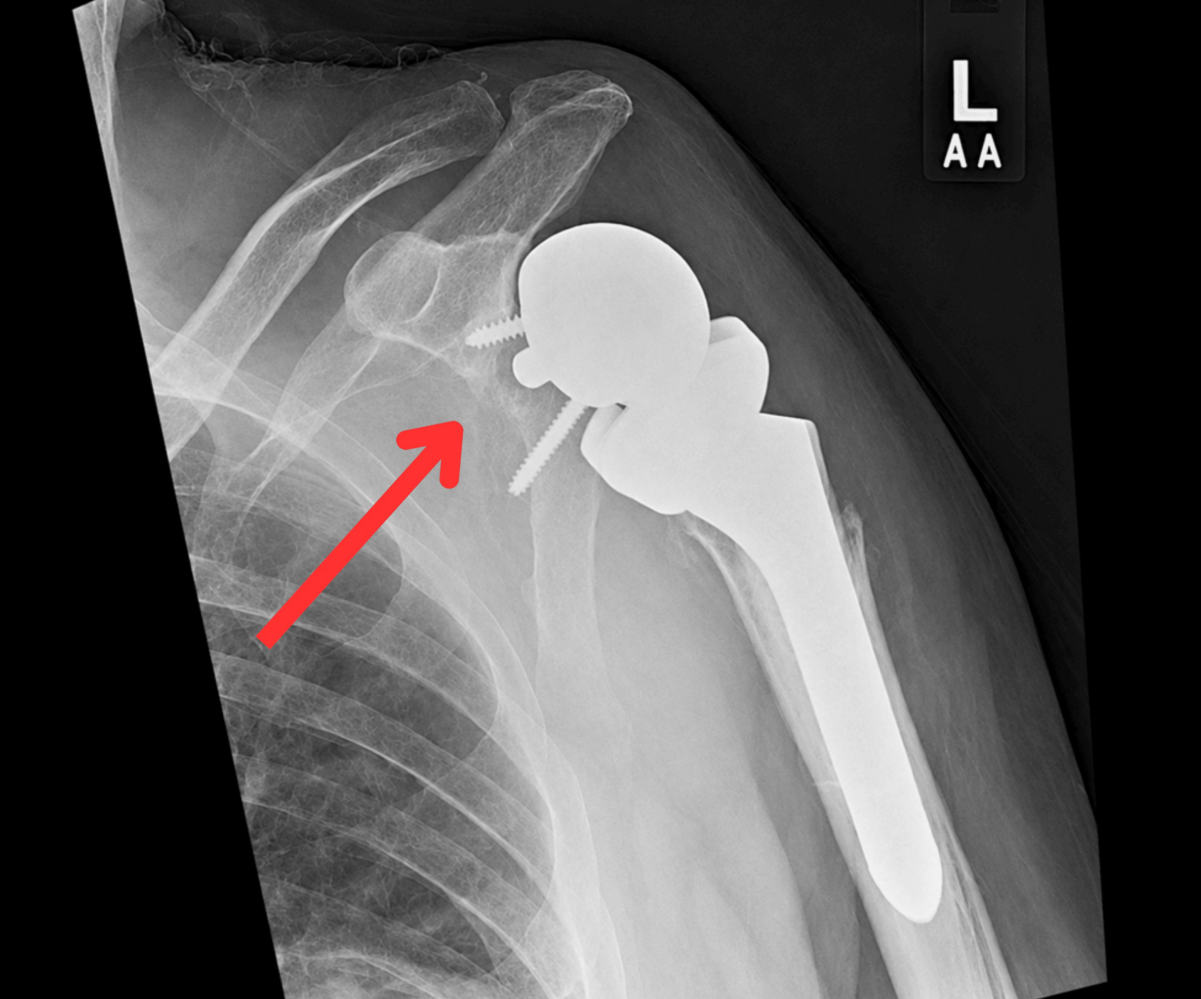
Follow-up radiograph

Radiographs obtained in December 2019 confirmed complete anterior dislocation of the reverse shoulder construct (Figure 3). 

**Figure 4:**
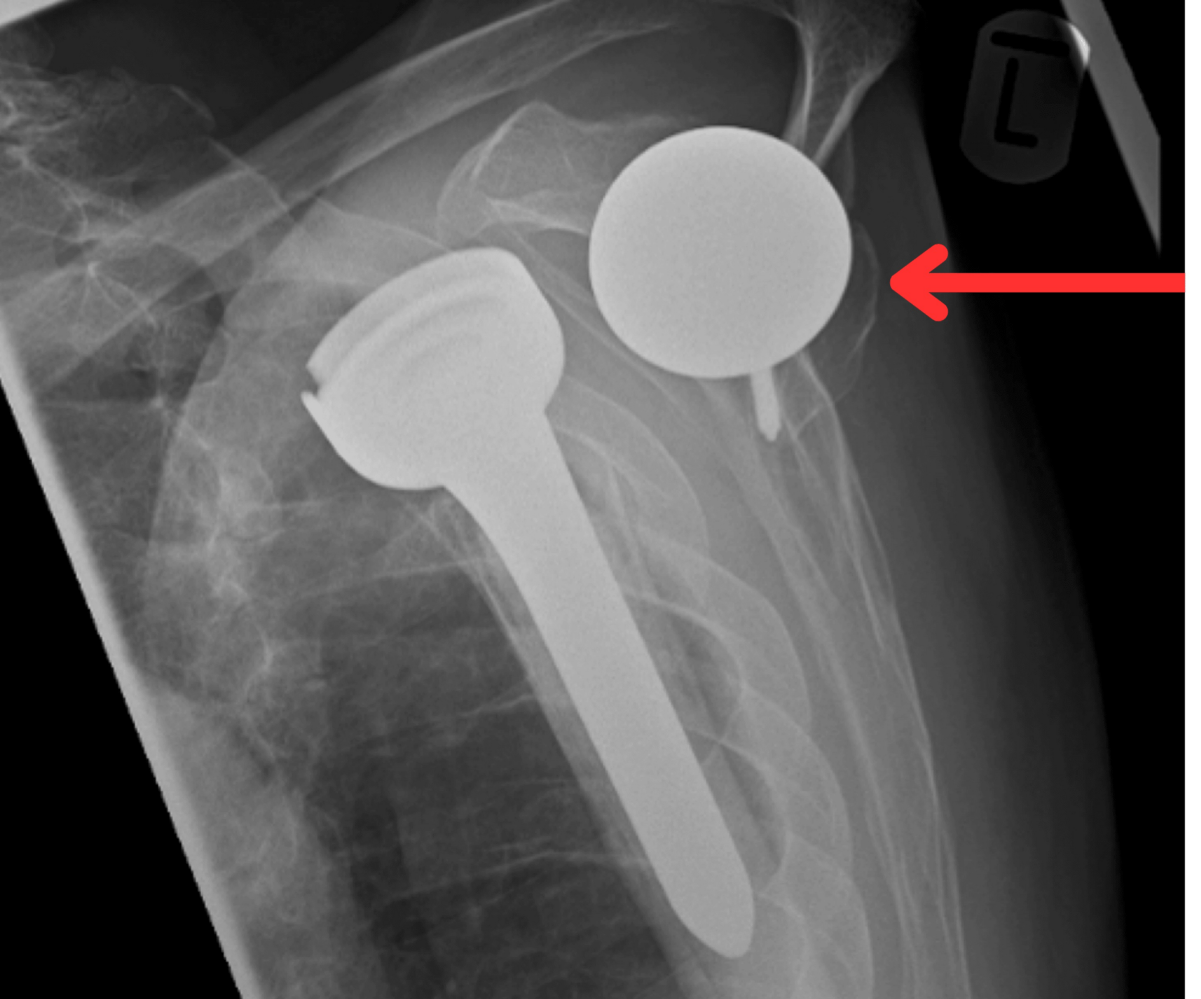
Second dislocation

Closed manipulation under anaesthesia was performed in December 2019. Post-reduction imaging demonstrated restoration of articulation and acceptable prosthesis position (Figure 4). He was discharged with a polysling with instructions to wear it continuously except for washing, and a staged physiotherapy plan was arranged.

**Figure 5:**
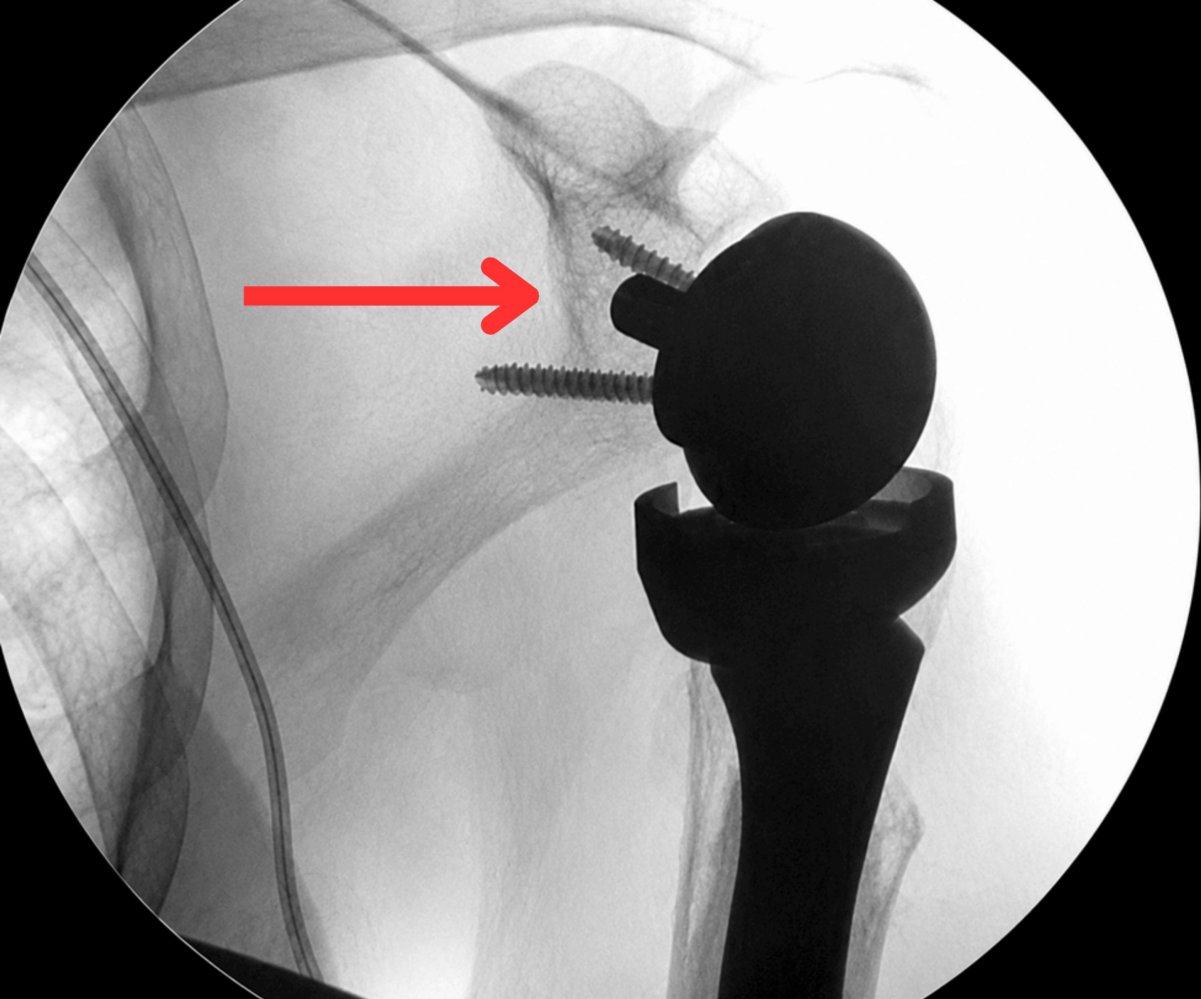
Intra-operative mobile image intensifier fluoroscopy

**Figure 6:**
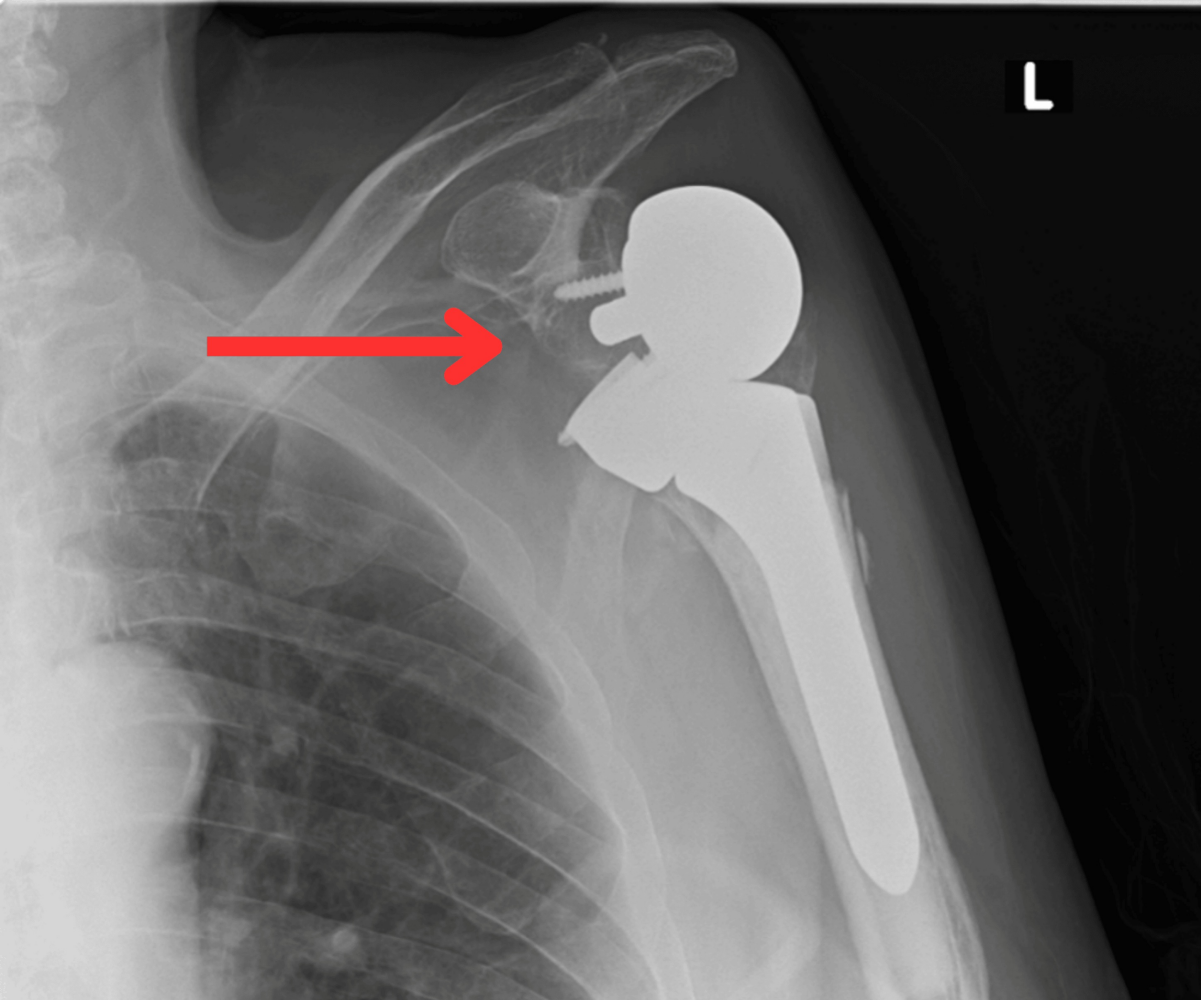
Post first open revision radiograph

Early follow-up radiographs in January 2020 showed a maintained reduction in the immediate post-manipulation period (Figures 5-6). However, during a subsequent physiotherapy attendance, recurrent dislocation was suspected clinically because of an abnormal shoulder contour and was confirmed radiologically. No definite traumatic event was identified. Neurological function was again documented as intact before revision surgery.

**Figure 7:**
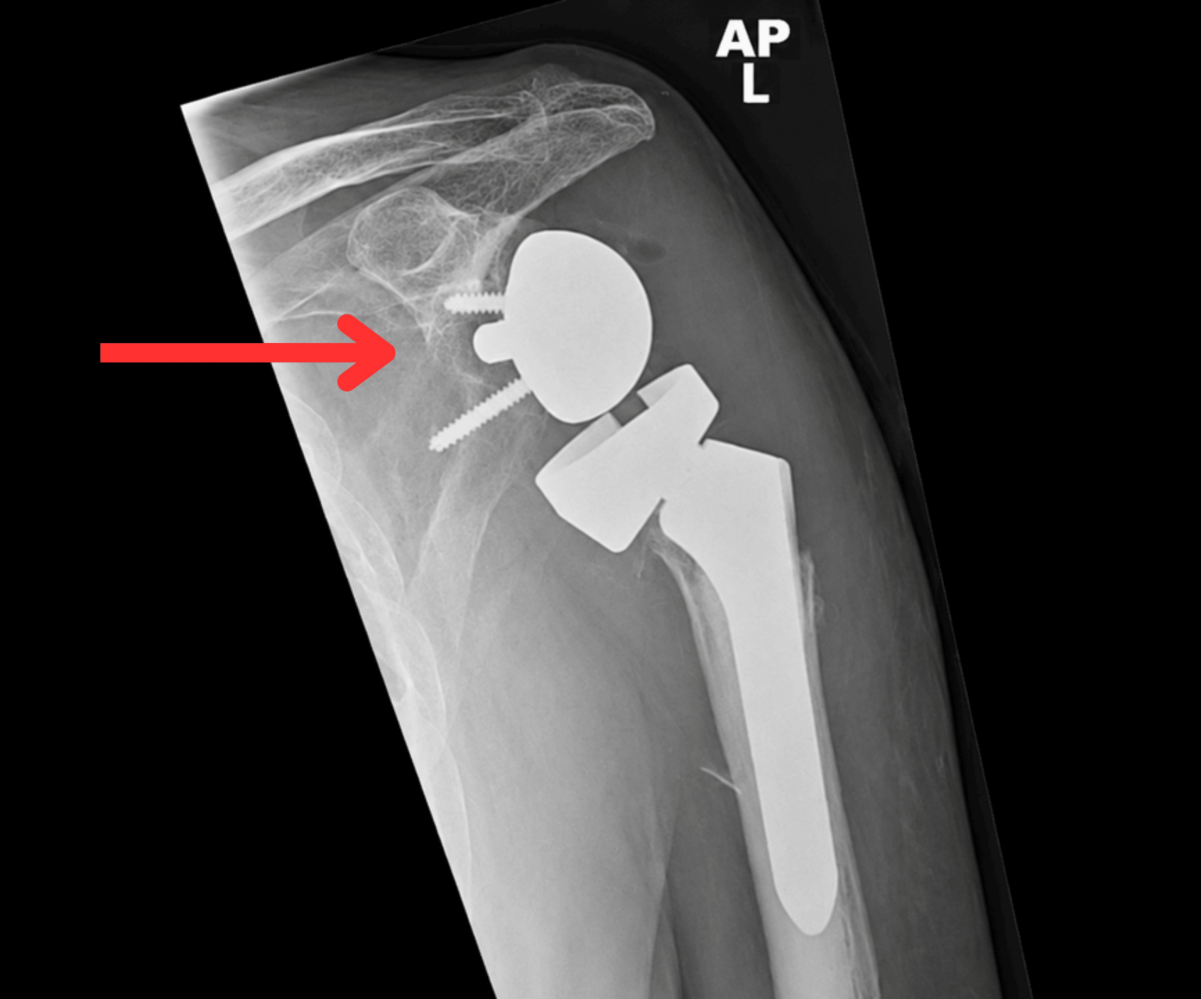
Post-operative radiograph following the first open revision

**Figure 8:**
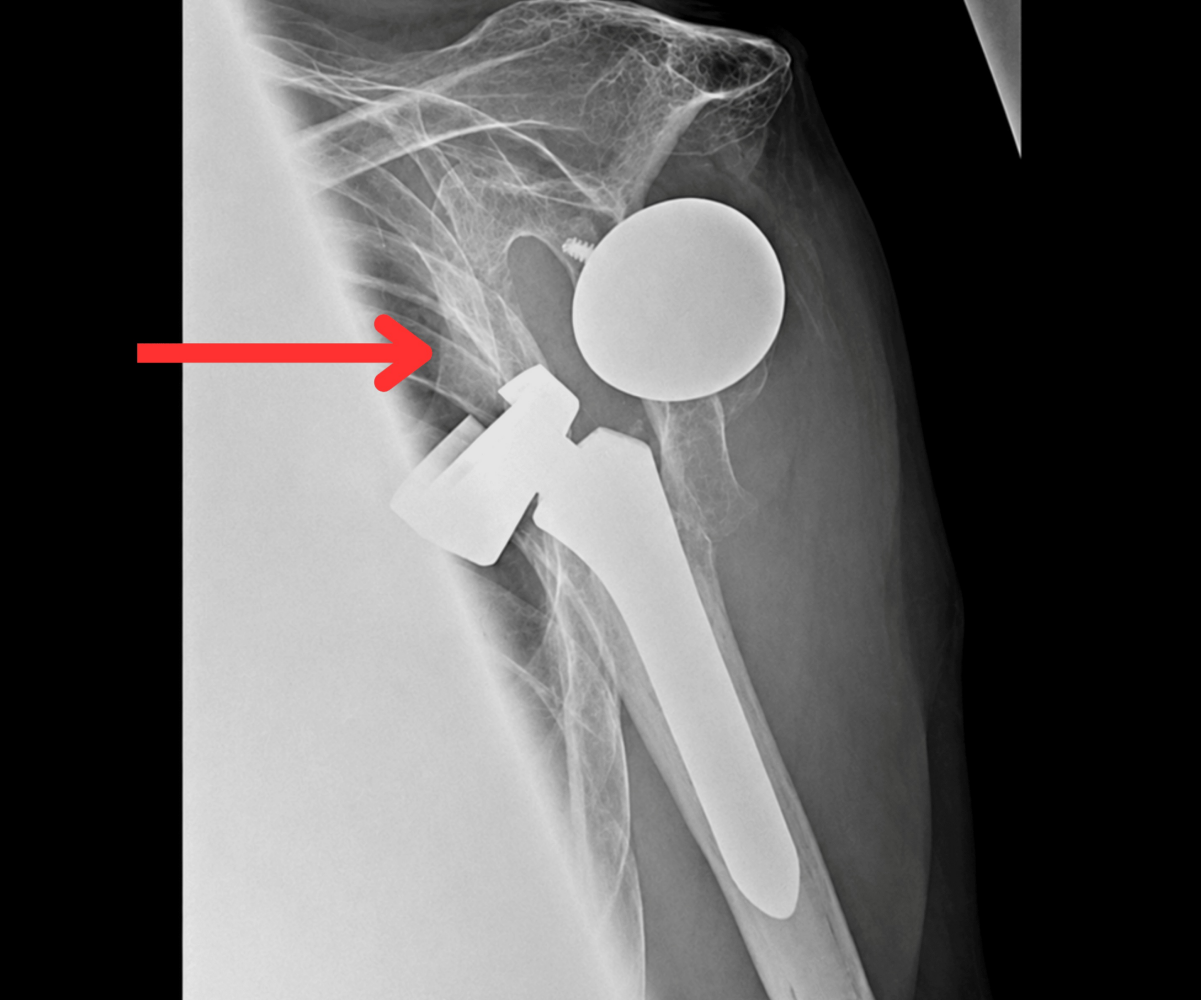
Radiograph confirming recurrent anterior dislocation prior to the second open revision

The first formal open revision aimed to address recurrent instability through implant-level correction. Intraoperative fluoroscopy was used to assess the construct and component alignment (Figure 7). The revision involved angle adjustment of the implant, liner exchange, and manipulation of the left elbow under anaesthesia for associated stiffness. Pre-revision and immediate post-revision radiographs documented the dislocated configuration and the temporary restoration of implant position after revision (Figure 8).

First open revision

During a subsequent physiotherapy session, the treating physiotherapist noted an abnormal shoulder contour and suspected recurrent dislocation, confirmed radiologically. The patient denied any specific traumatic episode. Intact neurological function in all peripheral nerve distributions was documented. Following discussion of risks and benefits, including infection, neurovascular injury, stiffness, persistent pain, re-dislocation, loosening, and intraoperative fracture, the patient consented to revision surgery.

The first formal open revision was performed in February 2020. This involved revision of the hybrid reverse polarity total shoulder replacement with adjustment of the angle of implant insertion, liner exchange, and manipulation of the left elbow under anaesthesia for associated elbow stiffness. The intra-operative image intensifier findings are shown below.

**Figure 9:**
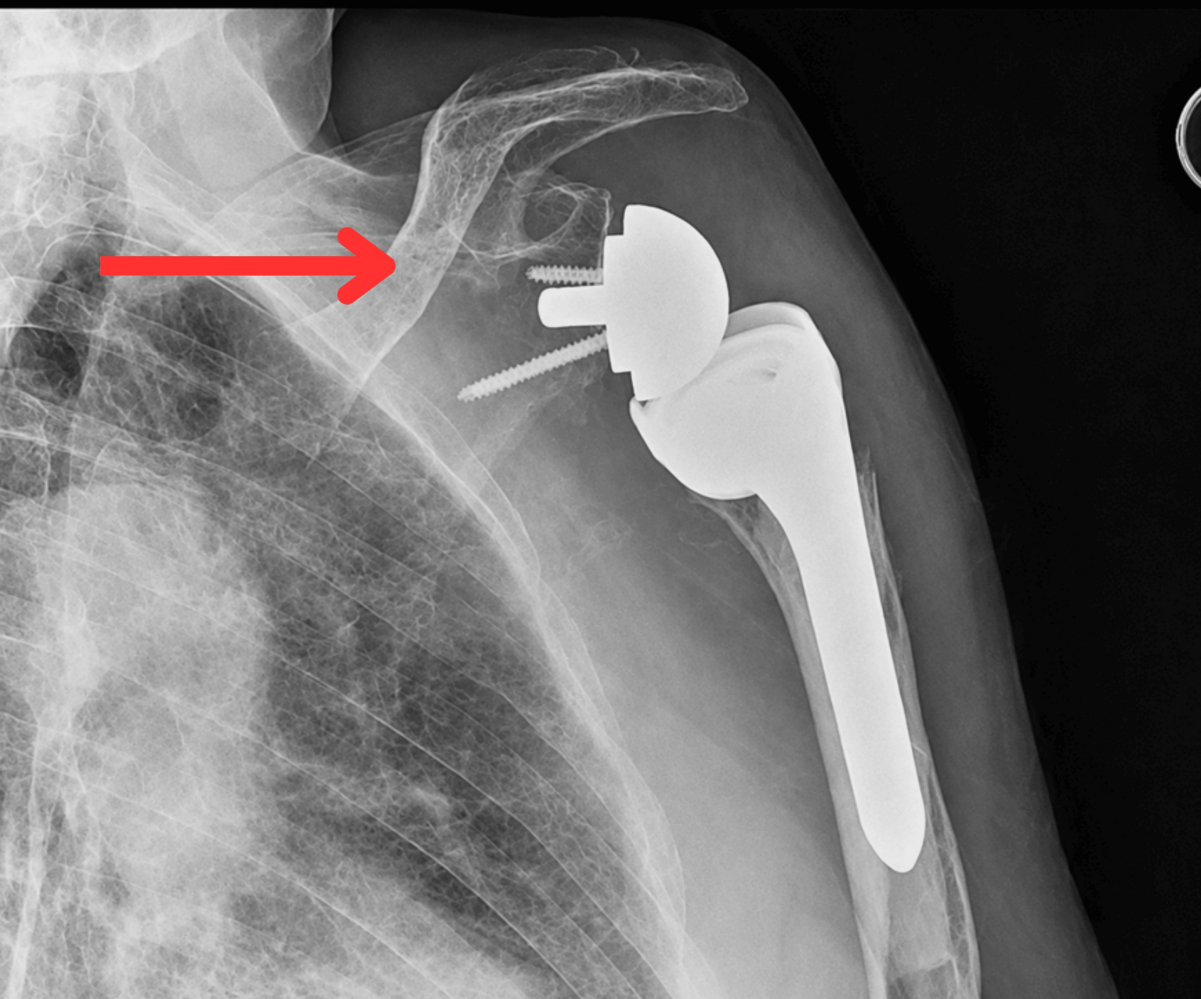
Immediate post-operative radiograph following the second open revision with glenosphere cup change

Figure 9 shows the post-operative radiographs following the first open revision (February 2020). Views demonstrate satisfactory implant positioning with the revised component angle and liner exchange in situ. Glenohumeral reduction is maintained in the immediate post-operative period.

Recurrent dislocation and second open revision: cup modification

During a subsequent physiotherapy attendance, the patient was again found to have a clinically and radiologically confirmed dislocation of the left reverse polarity shoulder replacement. He was admitted and underwent a further MUA in theatre; however, whilst still on the ward in the post-operative period, the shoulder re-dislocated. He was taken back to the theatre the following day for a further revision with a change of the glenosphere cup. Post-operative radiographs demonstrated immediate re-dislocation. The case was escalated and discussed via multidisciplinary team (MDT) with the Derby Shoulder Unit.

**Figure 10:**
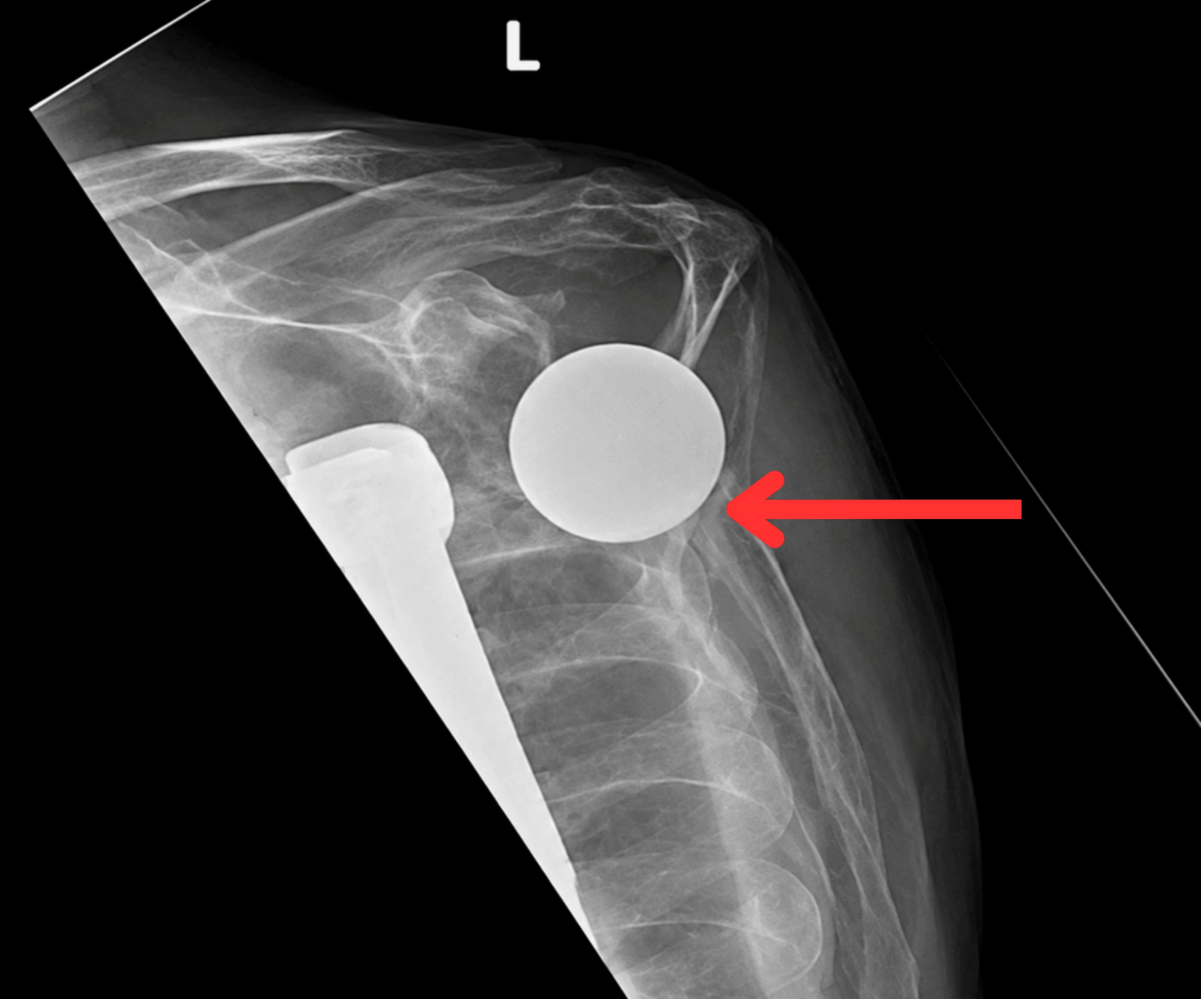
Follow-up radiographs

Figure 10 shows the radiographs confirming recurrent anterior dislocation prior to the second open revision (February 2020). Despite the previously performed liner exchange and angle adjustment, the prosthetic head has again dislocated anteriorly, with the humeral tray visible lateral to and displaced from the glenoid baseplate. This recurrence prompted escalation to MDT discussion with the tertiary shoulder unit.

Second open revision: glenosphere cup exchange

**Figure 11:**
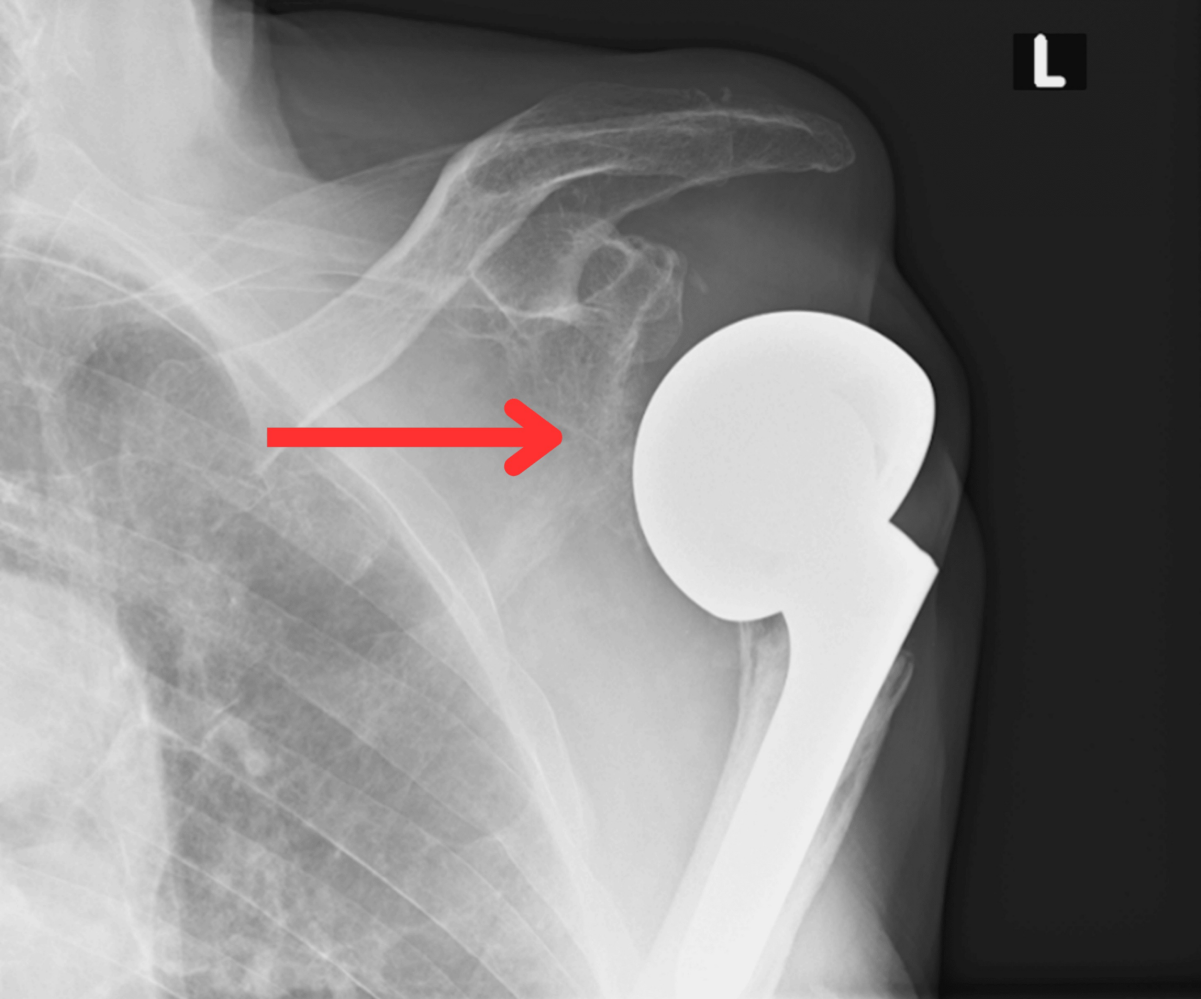
Post-operative radiograph following conversion to large-head bipolar hemiarthroplasty

Figure 11 shows the immediate post-operative radiographs following the second open revision with glenosphere cup change (February 2020). Two views demonstrate the revised construct. Despite the operative intervention, post-operative imaging confirmed persistent instability, with immediate re-dislocation documented on the ward.

**Figure 12:**
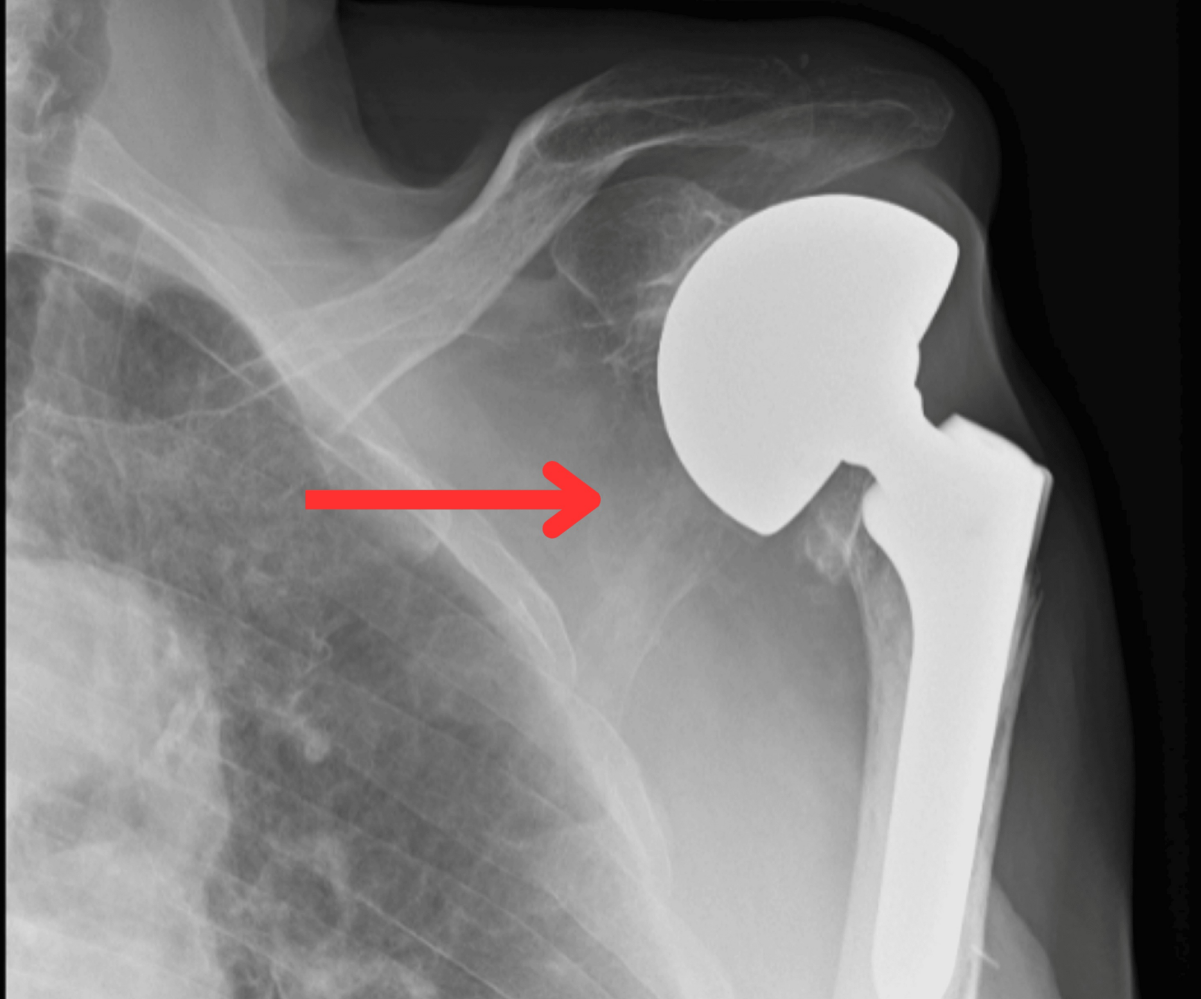
Additional post-operative radiographic views of the left shoulder bipolar hemiarthroplasty conversion

Figure 12 shows the follow-up radiographs in February 2020, demonstrating the position of the construct following the second open revision. Persistent dislocation is confirmed, with the humeral head displaced from the glenoid articular surface. All available implant-level revision options within the reverse polarity platform had at this stage been exhausted, prompting MDT consensus to convert to an alternative construct.

MDT decision and conversion to large-head bipolar hemiarthroplasty

Post-Conversion Radiographs: Large-Head Bipolar Hemiarthroplasty in Situ

Following MDT discussion with the specialist tertiary shoulder centre at Derby, a consensus was reached that the reverse polarity construct had been exhausted as a viable platform. Conversion to a large bipolar shoulder hemiarthroplasty was agreed as the most reasonable remaining option. It was acknowledged that near-normal function would not be achievable, but that this approach was anticipated to provide low-level functional improvement and potentially reduce further instability risk by eliminating the fixed-fulcrum RSA construct. The patient was counselled in full regarding limited functional expectations and the continued risk of dislocation.

Conversion of the hybrid reverse polarity total shoulder replacement to a bipolar shoulder hemiarthroplasty (left side) was performed with no intraoperative complications. Post-operative instructions included gentle hand-to-mouth and face range of motion only, with external rotation restricted to neutral. Physiotherapy-guided mobilisation was commenced, and the patient was discharged with a six-week outpatient follow-up arranged.

**Figure 13:**
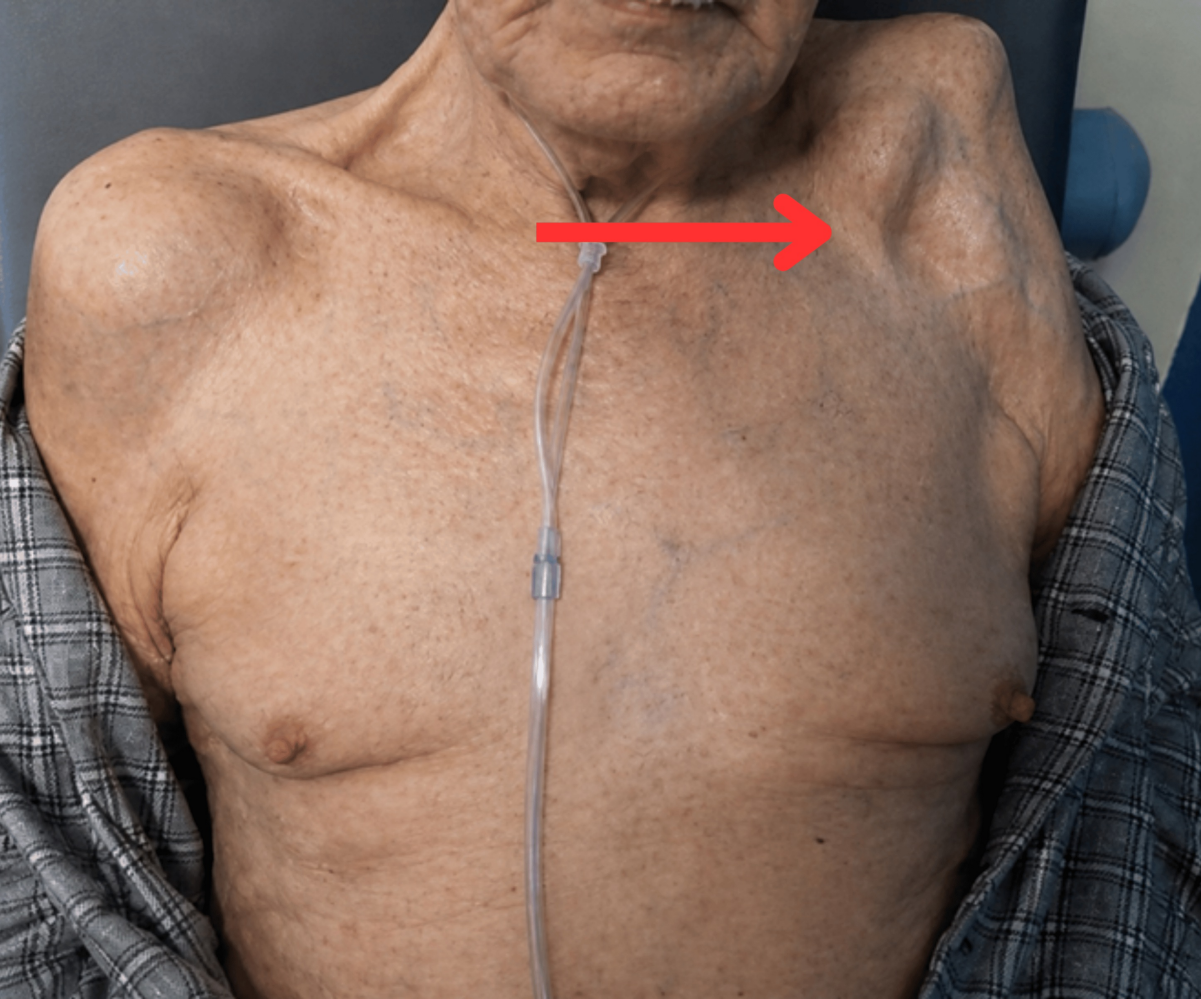
Anterior chest and bilateral shoulder view

Figure 13 shows the post-operative radiograph following conversion to large-head bipolar hemiarthroplasty. Anteroposterior (AP) view demonstrating the large-diameter bipolar humeral head component articulating against the residual glenoid surface. The stem is well-positioned within the humeral shaft. Note the absence of the glenoid baseplate, which was removed at conversion. The bipolar bearing mechanism is visible as the double-arc configuration of the humeral head component.

Dislocation of Bipolar Hemiarthroplasty and Brachial Plexus Neuropraxia

**Figure 14:**
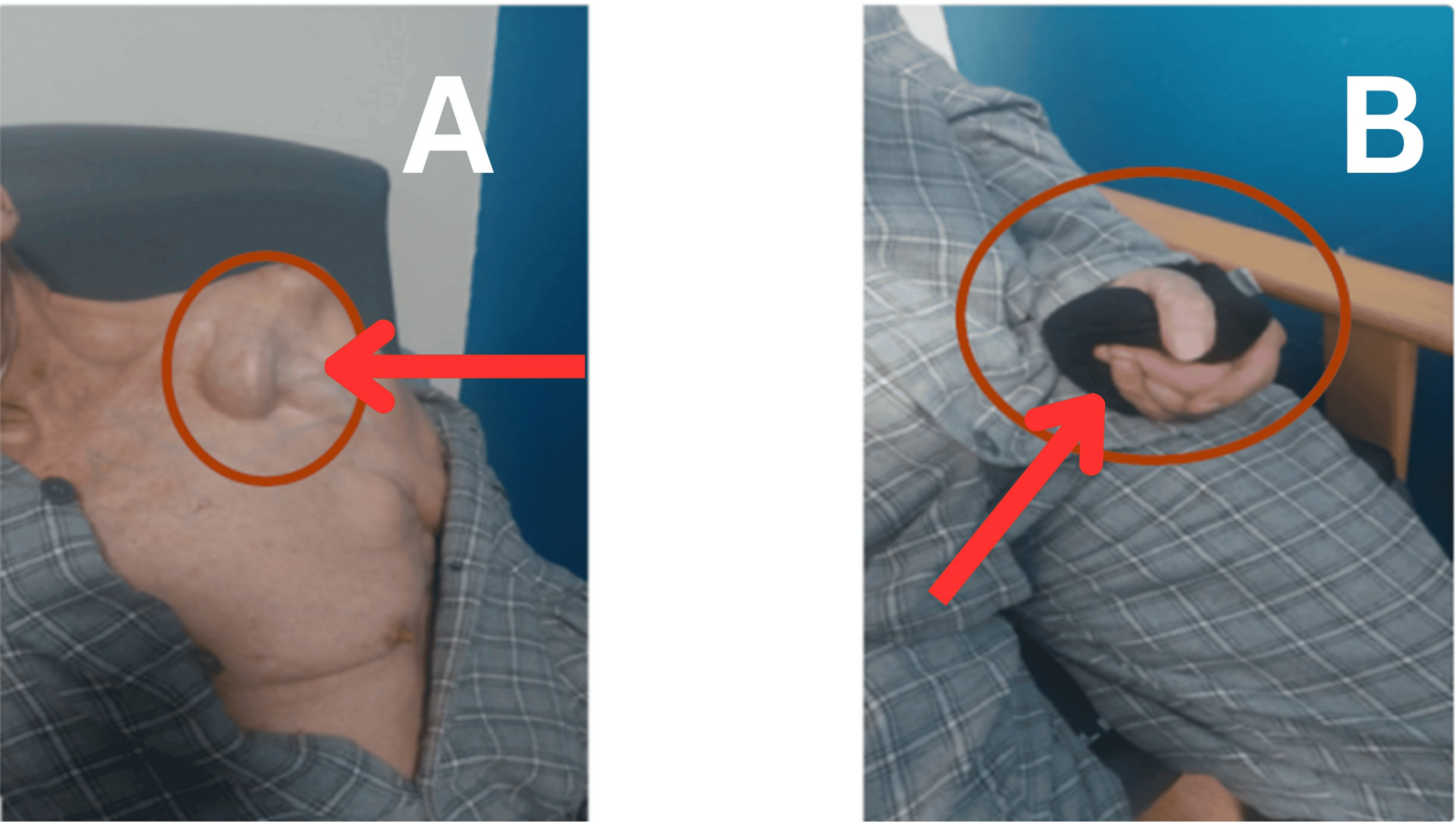
Close-up views of the left shoulder (A) and left hand (B)

Figure 14 shows the additional post-operative radiographic views of the left shoulder following bipolar hemiarthroplasty conversion. The large humeral head is centrally positioned, and early follow-up radiographs at this time point demonstrated satisfactory initial placement. The patient was reviewed in clinic with instructions for restricted range of motion and physiotherapy-supervised mobilisation.

Clinical Presentation: Dislocated Bipolar Implant With Brachial Plexus Neuropraxia

At subsequent outpatient review, the patient presented with recurrent left shoulder pain and dramatically impaired upper limb function. Clinical examination demonstrated that the bipolar hemiarthroplasty was frankly dislocated, with the large prosthetic head lying anteriorly in a subcutaneous position at approximately the level of the coracoid. Radiographs confirmed a complete anterior dislocation. Of critical concern, the patient reported complete inability to make a fist, hold objects, or perform any meaningful activity with the left hand. He had lost functional use of the entire left upper limb.

Clinical assessment revealed features consistent with brachial plexus neuropraxia secondary to direct mechanical impingement of the anteriorly migrated implant upon the plexus. The skin overlying the dislocated implant was intact with no evidence of breakdown. The bipolar bearing element appeared mechanically functional; rotation of the arm did not move the outer surface of the bipolar head, which is consistent with intact bipolar mechanics, though functional status was profoundly impaired.

**Figure 15:**
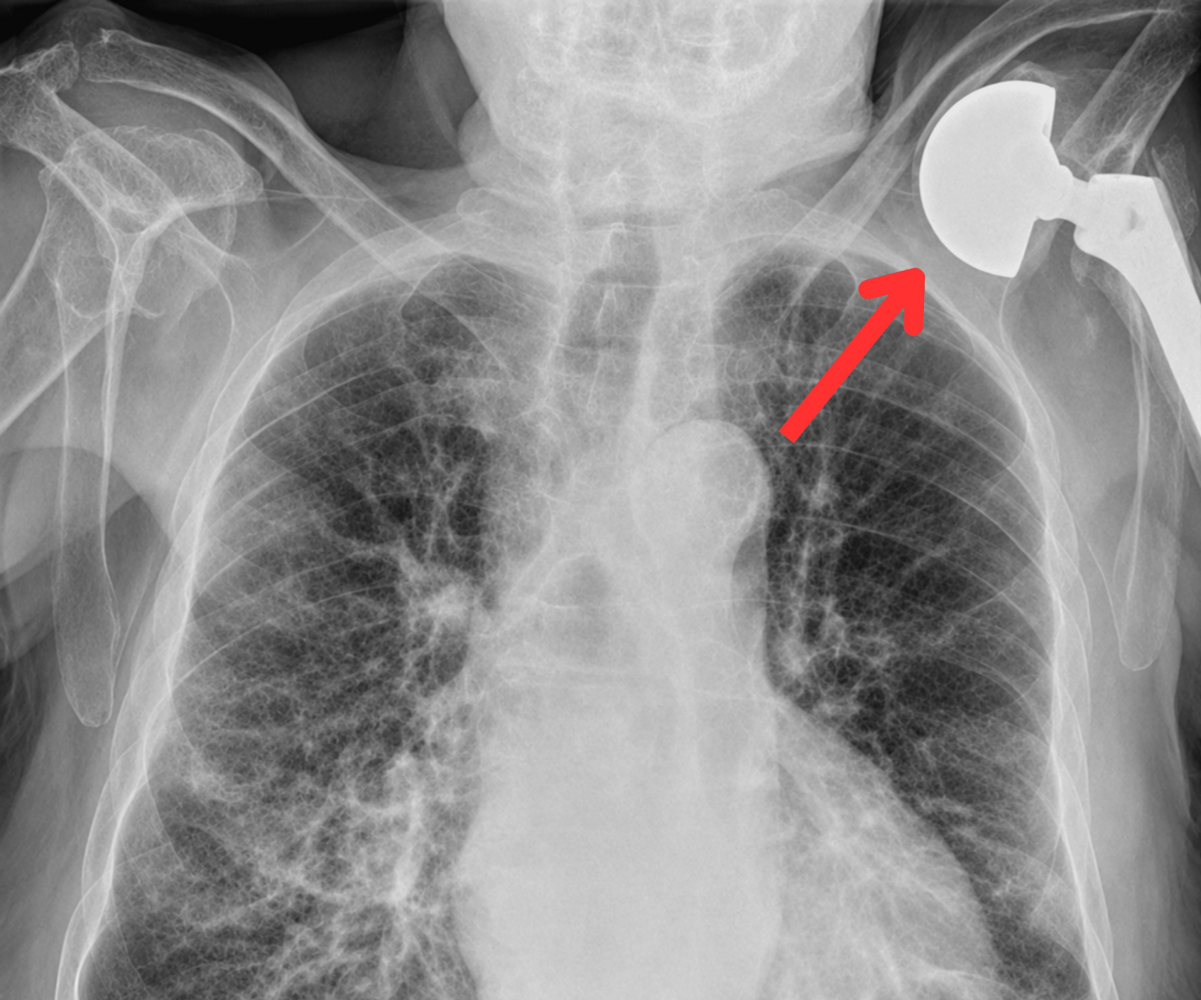
AP chest radiographs AP: anteroposterior

Figure 15 shows the anterior chest and bilateral shoulder view. Marked asymmetry is evident between the left and right shoulders. The left side demonstrates anterior displacement of the prosthetic humeral head, which is visible as a prominent subcutaneous convexity in the anterior deltopectoral region at the level of the coracoid. Loss of normal shoulder contour, acromion prominence, and relative shoulder elevation are consistent with a completely dislocated bipolar hemiarthroplasty. An oxygen delivery nasal cannula reflects the patient's profound medical comorbidity and frailty.

The anteriorly displaced prosthetic head is visible subcutaneously, with intact overlying skin and no wound breakdown. Right: Intrinsic-minus posturing of the left hand is consistent with lower brachial plexus (C8-T1) neuropraxia secondary to direct implant impingement on the plexus. The patient was unable to make a fist, grip objects, or perform any meaningful hand function. A functional resting hand splint is in situ.

Further surgical discussion and shared decision-making

The case was again subjected to MDT discussion. The remaining surgical option communicated to the patient involved complete explantation of all implant components, extensive proximal humeral bone grafting, and reconstruction using two large plates, carrying a very significant risk of infection, wound breakdown, hardware failure, re-fracture, and anaesthetic risk in the context of advanced COPD. Even in the event of technical success, functional outcomes would be limited to a hand-to-mouth range of motion only, with no capacity for functional external rotation, posterior reach, or activities of daily living.

Following a frank and thorough shared decision-making discussion, conservative management was accepted, acknowledging the dislocated position of the large bipolar head as the least harmful available option. The surgeon noted that the rounded bipolar head in situ would be tolerated in the dislocated position. Referral was subsequently made to Professor Jeys at the Royal Orthopaedic Hospital, Birmingham, to explore whether emerging solutions, including a Bayley-Walker prosthesis or a custom-linked prosthesis anchored to the scapular wing, might be feasible given the severely deficient glenoid and proximal humeral bone stock.

Subsequent complication: septic arthritis of the contralateral right shoulder

In February 2024, the patient, now with no functional left upper limb, presented to the Emergency Department with right shoulder pain, swelling, and restricted movement of one week's duration without precipitating trauma. Aspiration of the right shoulder and acromioclavicular joint yielded 70 ml of haemosanguineous fluid that grew gram-positive bacilli and cocci. He deteriorated clinically with low oxygen saturations and was transferred to the Resuscitation Unit for Sepsis-6 management. He underwent three formal right shoulder joint washouts. The third washout was followed by bloody discharge, raising concern for a vascular abnormality. A drain was inserted, inflammatory markers trended down, and the patient was declared fit for discharge under the care of Professor Neil Ashwood with antibiotics for concomitant infective exacerbation of COPD.

Histological analysis of tissue from washout raised the differential diagnosis of pigmented villonodular synovitis (PVNS) versus possible rheumatoid arthropathy. MRI and further haematological investigations were arranged. The patient was followed up in the fracture clinic for ultrasound assessment of the deltoid and MRI to exclude PVNS, with district nursing wound care arranged on discharge.

Late follow-up radiological surveillance: serial chest radiographs (2025)

Serial Chest Radiographs: Persistent Dislocated Bipolar Implant (February-April 2025)

Serial chest radiographs performed between February and April 2025 in the context of the patient's ongoing respiratory management (advanced COPD) provide important late radiological documentation of the dislocated bipolar implant. These images demonstrate the in situ position of the large humeral head component in the left axillo-deltopectoral region, with no evidence of hardware fracture, stem loosening, or progressive bony destruction. The right shoulder shows changes consistent with prior washout procedures.

Figure 15 shows the AP chest radiograph demonstrating dislocation of the left large-head bipolar hemiarthroplasty. The prosthetic head is displaced anterosuperiorly relative to its articulating surface, whilst the humeral stem remains seated within the humeral shaft without obvious radiographic evidence of loosening. The right shoulder demonstrates post-washout changes. The bilateral lung fields show a hyperinflation pattern consistent with advanced COPD, warranting ongoing respiratory monitoring.

Continued surveillance images confirm the stable position of the dislocated bipolar implant without progressive migration or implant fracture. These radiographs, obtained in the context of respiratory review, underscore the conservative management strategy accepted following shared decision-making and document the final documented state of the left shoulder arthroplasty in this patient's complex clinical journey.

## Discussion

Instability following reverse total shoulder arthroplasty for acute fracture

This case presents a sobering account of a catastrophic cascade of complications following an RSA performed in the acute fracture setting in an elderly, medically frail patient. Dislocation is one of the most feared complications of RSA, with reported rates in fracture-specific series ranging from 2.5% to 8.5% [3]. Patient-level risk factors include male sex, obesity, neurological impairment, prior shoulder surgery, and compromised soft tissue envelopes. Technical factors include inadequate glenosphere offset or inclination, insufficient humeral neck-shaft angle, and failure to restore deltoid tension. The early postoperative nature of the first dislocation, at one week during recommended ROM exercises, suggests that component positioning or soft tissue tensioning may have been suboptimal, though this cannot be concluded with certainty from the available records. Implant choice in the acute fracture setting also plays a role; hybrid cemented stems may behave differently from fully cementless stems in terms of subsidence risk, which could alter effective limb length and deltoid tension in the early post-operative period.

Revision surgery for recurrent dislocation

There is no universally accepted algorithm for managing recurrent dislocation following RSA. First-line intervention is closed reduction, with subsequent assessment of implant positioning. Where malposition is identified, revision surgery targeting version correction, glenosphere up-sizing, or liner exchange is recommended. In this case, two formal open revisions were performed, first adjusting the implant angle and exchanging the liner, then changing the cup, without achieving lasting stability. This suggests factors beyond simple mechanical malposition, including deltoid weakness secondary to COPD-related deconditioning, irreversible soft tissue incompetence, and patient factors compromising rehabilitation compliance.

Bipolar hemiarthroplasty as salvage

The concept of conversion to a large-head hemiarthroplasty as a last-resort salvage strategy in recurrently unstable RSA is recognised but uncommonly reported [5]. The rationale is that a large-diameter humeral head may be better contained within the remaining soft tissue sleeve without the fixed mechanical constraint of the RSA, thereby reducing dislocation risk. In this patient, however, the absence of sufficient glenoid bone stock and surrounding soft tissue integrity meant that even this construct dislocated, with far more severe consequences. The anterior migration of the large head to the level of the brachial plexus represents a complication that, to our knowledge, has been described only rarely and highlights the potential for devastating neurological injury in this specific failure mode. The serial chest radiographs documenting the persistent dislocated position five years after conversion represent a uniquely complete radiological documentation of this final state.

Emerging solutions and the role of multidisciplinary input

Multilevel failure of shoulder arthroplasty in the context of severely deficient glenoid bone stock presents a near-insoluble surgical problem. Referral to tertiary centres with expertise in complex revision or oncological reconstruction, as undertaken here via referral to the Royal Orthopaedic Hospital Birmingham, is essential. Custom-designed implants anchored to the scapular body, or linked megaprostheses analogous to those used in tumour surgery, represent potential future solutions in this rare scenario. The involvement of a regional MDT at multiple stages of this patient's care was commendable; however, earlier escalation to a specialist tertiary shoulder unit following the first failed revision might have altered the surgical trajectory.

Patient safety and informed consent

This case underscores the paramount importance of thorough, documented informed consent at each surgical stage. The patient was counselled regarding dislocation risk, nerve injury, and failure at each operation. That he continued to consent to sequential procedures in full knowledge of these risks reflects appropriate shared decision-making. The eventual transition to conservative management was equally a shared decision taken in the best interests of a patient who had exhausted available surgical options. The subsequent development of septic arthritis in the contralateral, and only functional, shoulder further illustrates the compounding nature of complications in medically frail, immunocompromised patients.

## Conclusions

We present a highly complex case of recurrent instability following reverse total shoulder arthroplasty for acute proximal humeral fracture in an elderly, medically frail patient, requiring closed reduction, two open revisions, and ultimately conversion to a large-head bipolar hemiarthroplasty, which itself dislocated and caused brachial plexus neuropraxia. The complete radiological timeline, from the index fracture in December 2019 through to serial chest radiographs in 2025 documenting the persistent dislocated implant position, represents an exceptionally comprehensive documentation of this clinical journey.

This case illustrates the importance of careful patient selection, meticulous surgical technique, early MDT involvement, and realistic patient counselling in the management of RSA for acute fracture in elderly, medically complex individuals. It highlights the profound consequences of recurrent implant failure and the current limitations of salvage strategies when glenoid and proximal humeral bone stock have been substantially compromised. Future developments in custom and tumour-analogue shoulder implants may offer solutions for this rare but devastating complication.
